# Frozen embryo transfer: Endometrial preparation by letrozole versus hormone replacement cycle: A randomized clinical trial

**DOI:** 10.18502/ijrm.v17i12.5793

**Published:** 2019-12-30

**Authors:** Alamtaj Samsami, Leila Ghasmpour, Sara Davoodi, Shaghayegh Moradi Alamdarloo, Jamshid Rahmati, Ali Karimian, Hamide Homayoon

**Affiliations:** ^1^ Infertility Research Center, Department of Obstetrics and Gynecology, School of Medicine, Shiraz University of Medical Sciences, Shiraz, Iran.; ^2^ Department of Anesthesiology, School of Medicine, Shiraz University of Medical Sciences, Shiraz, Iran.; ^3^ Department of Physiology, School of Medicine, Shiraz University of Medical Sciences, Shiraz, Iran.

**Keywords:** Letrozole, Hormone replacement, Endometrial, Preparation, Frozen, Embryo.

## Abstract

**Background:**

The endometrial preparation with stimulating natural cycles for frozen embryo transfer (FET) have benefits like lower cost and ease of use.

**Objective:**

Comparing the clinical outcome of letrozole versus hormone replacement (HR) for endometrial preparation in women with normal menstrual cycles for FET in artificial reproduction techniques.

**Materials and Methods:**

A total of 167 participants who had frozen embryos and regular ovulatory cycles were randomly divided into two groups for endometrial preparation. One group (82 women) was stimulated with letrozole 5mg/day and the other group (85 women) was hormonally stimulated by oral estradiol valerate (2 mg three times a day). All participants were followed serially by ultrasonography. Any patient who did not reach optimal endometrial thickness was excluded from the study. Implantation, biochemical and clinical pregnancy and abortion rate were reported.

**Results:**

There was no significant difference in the mean age, duration, and primary or secondary infertility, cause of the infertility, number, and quality of transferred embryos between the groups. The mean estradiol level on the day of transfer was 643 ± 217 in the HR group and 547 ± 212 in the letrozole group (P = 0.01), which was significantly different. The clinical pregnancy rate was 38.7 in the letrozole group, higher than the HR group (25.3) but not significantly different (P = 0.06).

**Conclusion:**

For endometrial preparation in women with a normal cycle, letrozole yields higher pregnancy rate although it is not significant; due to its cost, ease in use, and lower side effects, letrozole is a good choice.

## 1. Introduction

Frozen-thawed embryo transfer (FET) in in-vitro fertilization (IVF) cycles is a critical step to increase the rate of pregnancy and to prevent ovarian hyper stimulation syndrome (OHSS) (1).

Improved laboratory techniques by transferring fewer embryos in the fresh transfer cycles have led to an increase in the number of FET cycles (2). One of the main principles in FET is endometrial preparing for implantation of the thawed embryos (3). Points affecting FET outcome include: embryo quality, endometrium and embryo synchronization, and the reception of endometrial (4). Although increasing FET have significant effects on outcome, there is no accepted best protocol in women with ovulation for endometrial preparing (2).

There are different ways for endometrial preparation1) Artificial cycle FET (AC-FET): In this method, estrogen and progesterone are administered in a sequential regimen to mimic a normal endometrial cycle. At first, estradiol is initiated to induce proliferative endometrium and to suppress growing the dominant follicle. Estrogen is prescribed till endometrial thickness measured 7-9 mm on vaginal ultrasonography, then progesterone is initiated for making secretory phase (5). 2) Natural cycle (NC-FET): (A) The easiest method for preparing endometrium is production of steroids from a dominant follicle. Spontaneous LH surge or administering HCG determine the timing of embryo transfer (6). (B) Stimulated cycle FET: In this cycle, ovulation is induced by drugs such as clomiphene citrate, letrozole with or without HMG. The endometrium is prepared by endogenous estrogen and progesterone (7).

Letrozole is an aromatase inhibitor which does not decrease estrogen receptors. The normal central feedback system is not affected by letrozole and ovulation stays intact. It has no negative effect on endometrium (8).

Letrozole cause increasing FSH by not allowing estrogen making in granulosa cells from androgen. Its half-life is ± 2 days while clomiphene citrate half-life ± 2 wk; hence, antiestrogenic adverse effects of letrozole does not affected endometrium and cervix, and in comparison, to clomiphene citrate letrozole might have more ovulation induction properties like follicular growth and endometrial development (9). Therefore, mono ovulation with letrozole reduces the effect of high estradiol on the endometrium and embryo (10).

Considering the benefits, letrozole can be an effective endometrial preparing drug for FET cycles, its need more studies for clinical efficacy. The purpose of this study was to compare pregnancy outcome in stimulating with hormone replacement and endometrial preparation in patients with regular menstrual cycles with letrozole who were routinely candidate for the artificial cycle in our center.

## 2. Materials and Methods

### Setting

This study was conducted as a randomized clinical trial for a period of 12 months, commencing on January 2018. Women who were referred to the Infertility Center, Hazrate Zeinab Hospital, affiliated to Shiraz University of Medical Sciences were recruited in the study.

The inclusion criteria were women aged 18-42 yr, having frozen embryos, normal uterine cavity, normal endometrium without any endometrial polyp or sub-mucosal myoma (according to normal hysterosalpingography, saline infusion sonography or hysteroscopy), and BMI < 35 kg/m^2^. The exclusion criteria were other maternal medical disease and hydrosalpinx.

All participants meeting the inclusion criteria were divided into two groups based on the randomization table: the letrozole and hormone replacement (HR) groups. The sample size was calculated to be 74 cases in each group using the following formula with α = 0.05 and power 80%. Considering the probability of dropout, the study included 167 women, 82 in the letrozole group and 87 in the HR group. Two women from each group did not reach proper endometrial thickness and discontinued the intervention. Vaginal ultrasonography was done on the second day of the menstrual cycle for the evaluation of ovaries and endometrium.

### Endometrial preparation, embryo transfer, and luteal phase support

#### Letrozole group

Participants were prescribed 5 mg letrozole/day from the third day to the seventh day of the menstrual cycle. Follicular development was monitored by vaginal ultrasonography starting on the 10th day of the menstrual cycle; if the follicular diameter was ≥ 17 mm and the endometrial thickness reached 7-9 mm, they were given 10,000 unit of HCG, and 36-48 hr after that, they were progesterone ampule 100 mg was given intramuscularly/day for three to five days depending on the embryo's age. Then, the embryos were transferred and progesterone 100 mg/IM per day was continued for three days after the transfer and then changed to progesterone suppository 400 mg Q12 hr (rectal or vaginal) 14-16 days after the transfer.

If on the 10th day of the cycle, the dominant follicle was ≥ 14 mm to < 17 mm, a serial vaginal ultrasonography (every other day) was repeated without adding any medication until the follicle reached 17 mm or more or the endometrial thickness was 7-9 mm. After that, HCG was administered and like the first method progress was continued. However, if on the 10th day, the dominant follicle size was < 14 mm, HMG was injected daily for three to four days until the follicular diameter was > 17 mm or the endometrial thickness was 7-9 mm, and then HCG was injected. If by the 17th day of the cycle, the dominant follicle did not reach 17 mm or if the endometrial thickness was < 7 mm, the cycle was canceled.

#### HRT group

The participants were prescribed oral estradiol valerate (2 mg three times a day) starting on the second to the third day of menstrual cycle, and on the 10th day, the endometrial thickness was monitored by vaginal ultrasonography. If the endometrial thickness was 7-9 mm and three-line pattern, estrogen was continued and progesterone therapy was initiated 100 mg IM/day for three to five days depending on the embryo's age. Then, it was continued after the embryo transfer for three days and then changed to progesterone suppository 400 mg Q 12 hr.

If endometrial thickness was < 7 mm on the 10th day of the estradiol consumption, the estradiol dosage was increased to 8 mg/day and endometrium was evaluated by vaginal ultrasonography three to four days later. If the endometrial thickness was inappropriate, the cycle was canceled.

### Pregnancy diagnosis

HCG level was measured 14 days after the embryo transfer and if it was > 25 IU/L, it was defined as a biochemical pregnancy. Ultrasonography was performed 28-30 days after the embryo transfer, having a fetal heart beat was defined as a clinical pregnancy. For pregnant women in the letrozole group, progesterone suppository 400 mg twice daily was continued till 12 wk of gestational age. In the HR group, oral estradiol continued till 8 wk and suppository progesterone 400 mg twice daily was continued till 12 wk of gestational age.

### Ethical consideration

A written informed consent was obtained from all participants. The study protocol has been approved by the ethics committee of the Shiraz University of Medical Sciences (IR.SUMS.REC.1397.406).

### Statistical analysis

Date were analyzed using the Statistical Package for the Social Sciences, version 22, SPSS Inc, Chicago, Illinois, USA. The mean was compared using one-way analysis of variance (ANOVA) and two-sample t-tests. The proportion for the two groups was compared using the x2-test, p < 0.05 was considered to be statistically significant.

## 3. Results

This was a prospective trial comparing the outcome of endometrial preparation in frozen embryos transfer cycle by letrozole and hormone replacement. In the letrozole group, 82 women were followed, the analysis was limited to 80 women, since 2 of them (2.5%) were excluded due to non-development of the dominant follicle and endometrial growth (Figure I). Poor endometrial development had occurred in 2 out of 85 (2.3%) patients in the second group (HR). As shown in Table I, the two groups had no significant difference in age, BMI, time and type of infertility (primary or secondary), serum FSH, and AMH.

The cause of infertility (male factor, PCOS, unexplained, hypo-gonadotropic hypogonadism, tubal factor) with p = 0.22 has no significant difference.

Table II shows no significant difference between the stimulation duration, dose of gonadotropins, number of MII oocyte, and number of embryos.

As shown in Table III, there was no significant difference between the two groups pertaining to the duration of endometrial proliferation phase (day), the number of transferred embryo/cycles, the date of the embryo (cleavage or blastocyst), and the endometrial thickness at the end of follicular phase (mm).

The clinical pregnancy rate was higher in the letrozole group (38.7%) than in the artificial group (25.3%) but not significant. Furthermore, the first trimester abortion rate was non-significantly lower in the letrozole group (Table IV).

In this study, 20 patients who participated in the HR group and failed to reach pregnancy were scheduled to use letrozole in the next menstrual cycle, and of them, 35% became pregnant (7/ 20), but three had an early abortion (45% of clinical pregnancy).

**Table 1:** Demographic and hormonal data of study groups


	**HR group (n = 83)**	**Letrozole group (n = 80)**	**P-value**
Age	32.25 ± 4.6	33.28 ± 4.48	0.17*
BMI	26.5 ± 6.6	27.8 ± 8	0.72*
FSH	5.9 ± 2.3	5.9 ± 1.7	1*
AMH	2.8 ± 2.8	3 ± 2.09	0.69*
Time of infertility	7.5 ± 4.9	7.09 ± 4.1	0.51*
Primary/secondary infertility (yr)	54/29	61/19	0.11**
Data are presented as Mean ± SD, *Analysis was performed using the t-test and **Chi-square test			
HR: Hormone replacement; BMI: Body mass index; FSH: Follicle-stimulating hormone; AMH: Anti-Müllerian hormone

**Table 2:** Comparison of stimulation data and oocytes and embryos quality


	**HR group (n = 83)**	**Letrozole group (n = 80)**	**P-value**
Stimulation days	10.3 ± 2.4	11 ± 2.3	0.27
Human menopausal gonadotropin dose (IU)	2491 ± 795	2604 ± 901	0.87
No. of oocytes in metaphase II maturation stage (MII)	9.03 ± 5.79	9.07 ± 4.17	0.95
Number of embryos	4.58 ± 2.5	5.1 ± 2.2	0.16
Data are presented as Mean ± SD, Analysis was performed with the t-test.			
HR: Hormone replacement; IU: International unit, The quality of embryos in both groups had no significant differences (p = 0.72)

**Table 3:** Embryo transfer cycles data between case and control groups


	**HR group (n = 83)**	**Letrozole group (n = 80)**	**P-value**
Proliferation days	10.89 ± 1.95	10.83 ± 1.43	0.83
Endometrial thickness	8.26 ± 0.47	8.20 ± 0.58	0.44
Estradiol on the day of transfer	643 ± 217	547 ± 212	0.01
Number of embryo/cycles	2.48 ± 0.77	2.41 ± 0.66	0.58
Data are presented as Mean ± SD, Analysis was performed with the t-test, HR: Hormone replacement			

**Table 4:** Pregnancy outcome in letrozole and HR groups


	**HR group (n = 83)**	**Letrozole group (n = 80)**	**P-value**
Chemical pregnancy	28.9%	40%	0.13
Clinical pregnancy	25.3%	38.7%	0.06
Abortion	6%	7.6%	0.7
Data are presented as percentages; analysis was performed using the Chi-square test, HR: Hormone replacement			

**Figure 1:**
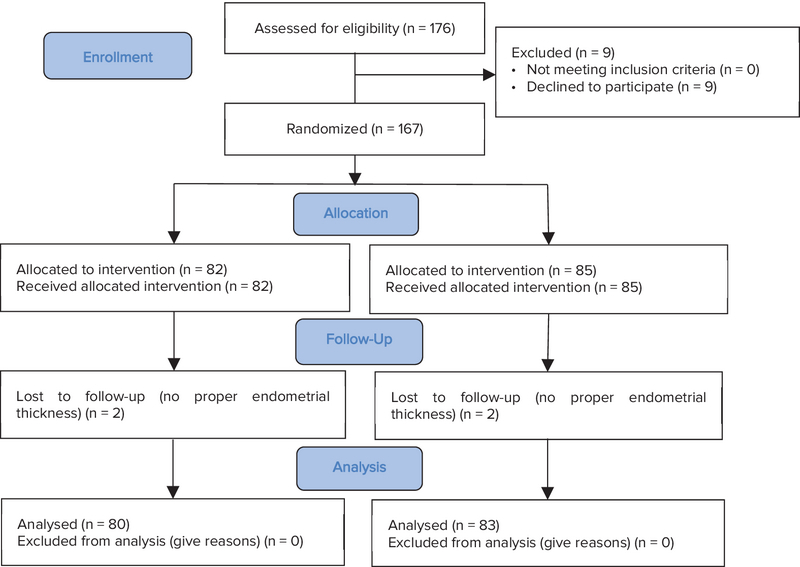
Consort diagram.

## 4. Discussion

In this study we detected that the clinical and chemical pregnancy rate was higher in the letrozole group although it was not significant. The study reported a significant lower level of estradiol in the letrozole group. We compared endometrial preparation for FET cycles with artificial hormone replacement versus mild ovarian stimulation with letrozole which did not have any significant difference in endometrial thickness between the two groups.

Studies reported that ovarian stimulation by aromatase inhibitors is because lower levels of estrogen production per follicle had overall lower estrogen level. Adding letrozole to gonadotropin for ovarian stimulation reduced the gonadotropin dosage and decreased the peak estradiol level (9). Sibai and colleagues study reported the mean endometrial thickness in the letrozole group was significantly lower (9.9 mm vs 9.1 mm), the clinical pregnancy rate in the letrozole group was significantly higher, but the chemical pregnancy rate had no significant differences like this study (3).

Developing corpus luteum and better endometrial receptivity led to higher pregnancy rate in the letrozole group. However, in the HR cycle, estrogen and progesterone does not suppress completely the pituitary gland and dominant follicle. If this follicle leads to spontaneous luteinization, the endometrium might have been exposed to progesterone earlier, which increases the risk of incorrect timing of the transfer, implantation, and pregnancy rate (11).

In Huang's study, the live birth rate was 38.89% and the clinical pregnancy rate was 47% in the letrozole FET cycle (12).

Letrozole rapidly absorbs and reaches maximum blood concentration 1 hr after its oral administration and also high bioavailability and a short half-life (45 hr), resulting in complete removal of the drug during the implantation period. The administration of letrozole at a low dose induces single follicular development and it has a high rate of ovulation; hence, preparing the endometrium for FET.

When considering the advantages of endometrial preparation methods, pregnancy outcome is not the only important issue, but one has to consider its convenience, cost, and lower risk of venous thromboembolic events in obese and high-risk patients.

Artificial hormonal cycles might still be the first choice for women in FET cycles, but this protocol cause cost and discomfort. It requires administration of high estrogen and progesterone doses for luteal phase support in the first trimester. So, in patients with normal menstrual cycles, it is better to find other choices.

There has been no protocol for endometrial preparing by letrozole.

## 5. Conclusion

For endometrial preparation in women with a normal cycle, letrozole yields higher pregnancy rate although it is not that significant; due to its benefit, lower side effects, and the patient's comfort in the use of medication, letrozole is a better choice for endometrial preparation.

##  Conflict of Interest

The authors declare that they have no conflict of interests.
